# Solid tumours showing oligoprogression to immune checkpoint inhibitors have the potential for abscopal effects

**DOI:** 10.1007/s11604-023-01516-w

**Published:** 2023-12-14

**Authors:** Makoto Ito, Souichiro Abe, Sou Adachi, Yukihiko Oshima, Arisa Takeuchi, Wataru Ohashi, Takashi Iwata, Tetsuya Ogawa, Akiko Ota, Yasuaki Kubota, Takahito Okuda, Kojiro Suzuki

**Affiliations:** 1 Department of Radiology, Aichi Medical University Hospital, 1-1 Yazako-Karimata, Nagakute, Aichi 480-1195 Japan; 2 Department of Radiation Oncology, Anjo Kosei Hospital Aichi Prefectural Welfare Federation of Agricultural Cooperatives, 28 Higashihirokute, Anjo-Cho, Anjo, Aichi 446-8602 Japan; 3 Department of Biostatistics, Clinical Research Center, Aichi Medical University, 1-1 Yazako-Karimata, Nagakute, Aichi 480-1195 Japan; 4 Department of Oncology Center, Aichi Medical University Hospital, 1-1 Yazako-Karimata, Nagakute, Aichi 480-1195 Japan; 5 Department of Otorhinolaryngology-Head and Neck Surgery, Aichi Medical University Hospital, 1-1 Yazako-Karimata, Nagakute, Aichi 480-1195 Japan; 6 Department of Oncology, Toyota Memorial Hospital, 1-1-1 Heiwa-Cho, Toyota, Aichi 471-8513 Japan; 7 Department of Urology, Toyota Memorial Hospital, 1-1-1 Heiwa-Cho, Toyota, Aichi 471-8513 Japan; 8 Department of Radiation Oncology, Toyota Memorial Hospital, 1-1-1 Heiwa-Cho, Toyota, Aichi 471-8513 Japan

**Keywords:** Abscopal effect, Immune checkpoint inhibitors, Oligoprogressive disease, Oligometastasis, Radiotherapy, PD-L1

## Abstract

**Purpose:**

Given the uncertainty surrounding the abscopal effect (AE), it is imperative to identify promising treatment targets. In this study, we aimed to explore the incidence of AE when administering radiotherapy to patients with oligoprogressive solid tumours while they are undergoing treatment with immune checkpoint inhibitors (ICIs).

**Materials and methods:**

In this multicentre prospective observational study, oligoprogressive disease was defined as a < 20% increase in lesions compared to > 2 months before enrolment. We enrolled patients who requested radiotherapy during the ICI rest period between 2020 and 2023. AE was considered present if ≥ 1 non-irradiated lesion decreased by ≥ 30% before the next line of systemic therapy started.

**Results:**

Twelve patients were included in this study; the common primary lesions were in the lungs (four patients) and kidneys (three patients). AEs were observed in six (50%) patients, with a median time to onset of 4 (range 2–9) months after radiotherapy. No significant predictors of AEs were identified. Patients in the AE group had a significantly better 1-year progression-free survival (PFS) rate than those in the non-AE group (*p* = 0.008). Two patients from the AE group were untreated and progression-free at the last follow-up. Four (33%) patients experienced grade 2 toxicity, with two cases attributed to radiotherapy and the other two to ICI treatment. No grade 3 or higher toxicities were observed in any category.

**Conclusion:**

Patients with oligoprogressive disease may be promising targets with potential for AEs. AEs can lead to improved PFS and, in rare cases, to a certain progression-free period without treatment.

**Secondary Abstract:**

Irradiating solid tumours in patients with oligoprogressive disease during immune checkpoint inhibitor therapy may be a promising target with the potential for abscopal effects (AEs). AEs can lead to improved progression-free survival and, in rare cases, to a certain progression-free period without treatment.

## Introduction

With the advent of immunotherapy, the prognosis of patients with advanced-stage cancer has considerably improved. Immune checkpoint inhibitors (ICIs) are typical examples of such drugs, and their use has expanded recently to treat various solid tumours [1]. However, favourable responses are limited to a small number of patients, and the emergence of resistance to ICIs is a significant concern [2, 3]. If the effect is poor, the next line of treatment is considered. However, there are frequently no effective treatment options, especially for frail patients. Therefore, activating antitumour immunity through additional treatments is currently the focus of attention. This strategy is exemplified by a combination of systemic therapies that reprogram the immune microenvironment of tumours [4, 5]. Another option is to combine ICI therapy with radiotherapy.

Although radiotherapy is a local treatment, it is also used to relieve symptoms and reduce the overall tumour burden in advanced cases. Simultaneously, the abscopal effect (AE), though rare, is a phenomenon where antitumour immunity is induced, leading to the regression of tumours outside the irradiated area [6]. Improved prognosis is a result of the AE and is highly beneficial for patients [7, 8]. However, the AE has been criticised for its unpredictable occurrence. Numerous reports have shown few or no AEs resulting from the combination of ICI and radiotherapy [9–12]. Radiotherapy imposes a time and cost burden on the patient and sometimes severe toxicity. Therefore, targeting AEs and using radiotherapy extensively without the goal of pain relief or local control are inappropriate. It is necessary to identify promising targets for which the AE can be expected to have a high probability of success.

In this study, we focused on a condition called oligoprogressive disease. Oligoprogressive disease is not clearly defined; however, it refers to patients with slow or limited progression during systemic treatment [13]. Although patients with oligoprogressive disease are at an advanced stage, local treatments, such as surgery and radiotherapy, may improve prognoses [14]. We hypothesised that patients with oligoprogressive disease who received radiotherapy during ICI treatment would have a high rate of AEs. This prospective multicentre observational study aimed to investigate the incidence of AEs based on the hypothesis.

## Materials and methods

### Patients

We conducted a multicentre prospective observational study of patients who underwent radiotherapy between November 2020 and January 2023. The cohort comprised patients with solid tumours that were oligoprogressive in response to ICI administration. No clear consensus existed on the definition of oligoprogressive disease when this study was designed. Therefore, oligoprogressive disease was defined as a < 20% increase in lesions compared to > 2 months before enrolment, which implies categorising it within the stable disease category. Other eligibility criteria were as follows: (1) age at enrolment ≥ 20 years; (2) Eastern Cooperative Oncology Group performance status of 0–2; (3) expected survival ≥ 3 months; (4) diagnosis of solid tumour (excluding haematological disease) and receiving ICI; (5) irradiated target with a long (total) diameter ≥ 3 cm; and (6) > 1 lesion, other than the irradiated target, being evaluated. The exclusion criteria were as follows: (1) multiple cancers (concurrent overlapping cancers or iatrogenic overlapping cancers with a disease-free interval of ≤ 5 years); (2) stroke, cerebral haemorrhage or myocardial infarction within 6 months before enrolment; (3) autoimmune disease (excluding type I diabetes, manageable thyroid disease and skin disease); (4) use of immunosuppressive drugs or adrenal corticosteroids (prednisolone equivalent ≥ 30 mg/d); (5) measurable lesions after radiotherapy were brain lesions only or skin lesions only; (6) planned to discontinue ICI after radiotherapy; and (7) history of irradiation at the same site as the present case (re-irradiation). Patients who requested radiotherapy and met all inclusion criteria and none of the exclusion criteria were included in the analysis. As this was an exploratory observational study, the study size was determined based on the number of patients expected to be included in the timeframe.

This study was approved by the Ethics Committee of Aichi Medical University Hospital in Japan (application number 2020–147), Anjo Kosei Hospital, and Toyota Memorial Hospital with an opt-out approach regarding the analysis before this study. This study was conducted in accordance with the tenets of the Declaration of Helsinki and its subsequent amendments.

### Immunotherapy and radiotherapy

ICIs included anti-programmed cell death-1 (PD-1) antibodies (pembrolizumab and nivolumab) as a mainstay. Anti-PD-1 antibodies were administered alone or in combination with other types of ICIs or systemic therapy. Any number of pretreatment systemic therapy lines could be used; however, as stated in the eligibility criteria, at least 2 months had to elapse between the start of ICI administration and that of radiotherapy. The treatment schedule followed a standard regimen for each disease. If grade 2 toxicity was observed according to the Common Terminology Criteria for Adverse Events (CTCAE), the administration was suspended until the patient recovered to grade 1. We terminated administration in cases of serious adverse events, such as grade ≥ 3 toxicity or confirmed overt progressive disease (PD), according to Response Evaluation Criteria in Solid Tumours (RECIST) version 1.1 [15].

Radiotherapy was administered during the rest period, ensuring avoidance of concurrent ICI administration. We used 6- or 10-MV X-rays from linear accelerators in all cases. Although dose fractionation was determined by the physician in charge, considering clinical information, such as target size and location, all patients underwent hypofractionated radiotherapy to ensure that irradiation was completed during the rest period. Patients were treated with three-dimensional conformal radiation therapy or intensity-modulated radiation therapy.

### Outcome evaluation

The primary endpoint was the rate of the AE. An AE was considered present if at least one of the non-irradiated lesions decreased by at least 30% by the time the disease was classified as PD (i.e. before the next line of systemic therapy began). The secondary endpoints were irradiation completion rate, local effect, 1-year overall survival (OS) rate, 1-year progression-free survival (PFS) rate and toxicity rate. Local effects of the irradiated area were assessed objectively by a physician based on RECIST after 3 months (range: 2–4 months), and those with a complete response (CR) or partial response (PR) were considered responders. In addition, patients subjectively rated whether there was an improvement in pain relief. Toxicity was graded according to CTCAE version 5.0. We measured the time from the date of initiation of radiotherapy to the event. Programmed cell death ligand 1 (PD-L1) expression was classified according to the tumour proportion score as strongly positive (≥ 50%), positive (1–49%), negative (< 1%) or unknown. These classifications were based on existing data measured on explanted specimens or biopsy results, irrespective of the site to be irradiated. Only one patient had received systemic therapy before sample collection.

### Statistical analyses

All statistical analyses were performed using EZR version 1.55 (Saitama Medical Center, Jichi Medical University, Saitama, Japan) based on R and R Commander [16]. Patient characteristics were compared using Fisher’s exact test for categorical variables and Student’s t-test or the Mann–Whitney *U* test for continuous variables. Certain quantitative variables were grouped and analysed as categorical variables. Additionally, the Kaplan–Meier method was used to estimate survival rates, and comparisons were performed using the log-rank test. Statistical significance was set at *p* < 0.05.

## Results

### Primary endpoint outcomes and patient characteristics

**Fig. 1:**
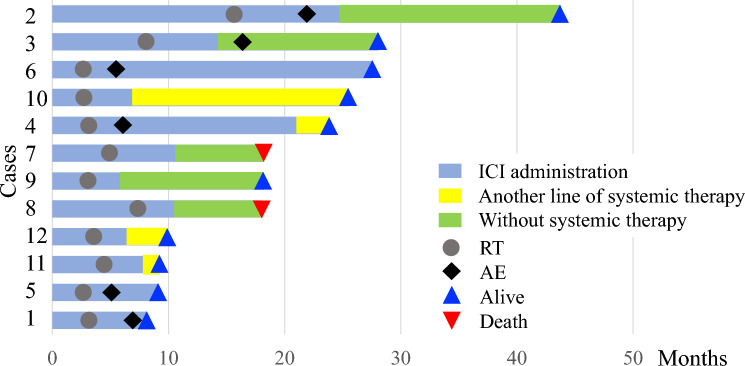
Clinical course of each patient, starting with the administration of the ICI. *ICI* immune checkpoint inhibitor, *RT* radiotherapy, *AE* abscopal effect

Twelve Japanese patients met the inclusion criteria, of whom six (50%) had AEs. The median time from irradiation to AE confirmation was 4 (range 2–9) months. Table 1 summarises patient characteristics grouped according to the presence or absence of the AE. The most common primary disease was lung cancer, and the histological type was adenocarcinoma, with bone and lymph node irradiation for pain relief in most cases. The univariate analysis revealed no factors that significantly contributed to the occurrence of the AE. For immunohistochemistry of PD-L1, the 22C3 pharmDx assay was used, except in one melanoma case for which the 28–8 pharmDx assay was used. All three patients with strongly positive PD-L1 expression had AEs, whereas the three patients with negative PD-L1 expression did not. The AE group had predominantly male (*p* = 0.061) patients and had higher baseline white blood cell counts (*p* = 0.093). Table 2 shows details of the case-specific PD-L1 expression and treatment. The clinical course of each patient, starting with the administration of the ICI, is shown in Fig. 1.

**Table 1:** Patient characteristics grouped by the presence or absence of the abscopal effect

	AE + (*n* = 6)	AE- (*n* = 6)	*p*
Median age in years (range)	71 (47–83)	66 (31–76)	0.630
*Sex*			0.061
Male	6 (100%)	2 (33%)	
Female	0 (0%)	4 (66%)	
*Performance status*			0.999
0	4 (66%)	4 (66%)	
1	1 (17%)	2 (33%)	
2	1 (17%)	0 (0%)	
*Primary sites*			0.766
Lung	3 (50%)	1 (17%)	
Kidney	1 (17%)	2 (33%)	
Head and neck	1 (17%)	1 (17%)	
Others	1 (17%)	2 (33%)	
*Pathology*			0.766
Adenocarcinoma	3 (50%)	1 (17%)	
Clear cell carcinoma	1 (17%)	2 (33%)	
Squamous cell carcinoma	1 (17%)	1 (17%)	
Others	1 (17%)	2 (33%)	
*PD-L1 expression (tumour proportion score)*			0.156
Strongly positive (≥ 50%)	3 (50%)	0 (0%)	
Positive (1–49%)	1 (17%)	1 (17%)	
Negative (< 1%)	0 (0%)	3 (50%)	
Unknown	2 (33%)	2 (33%)	
*Blood sampling data at registration*			
White blood cell count (/μl)	7750 (4800–11000)	4550 (2900–8200)	0.093
Neutrophil (/μl)	5369 (3768–7979)	3179 (1430–6708)	0.132
Lymphocyte (/μl)	1093 (485–3124)	991 (608–1268)	0.699
CD4/CD8 rate	1.71 (0.63–3.27)	1.73 (1.27–3.65)	0.548
Total protein (g/dL)	7.0 (6.3–7.6)	7.1 (6.0–7.8)	0.872
Albumin (g/dL)	3.6 (2.3–4.3)	3.9 (3.2–4.4)	0.422
C-reactive protein (mg/dl)	1.34 (0.19–19.63)	0.245 (0.01–4.27)	0.485
*Number of ICI lines*			0.999
1	4 (66%)	3 (50%)	
2	1 (17%)	1 (17%)	
≥ 3	1 (17%)	2 (33%)	
*Current ICI agent*			0.999
Nivolumab (monotherapy)	2 (33%)	3 (50%)	
Nivolumab (combined therapy)	1 (17%)	0 (0%)	
Pembrolizumab (monotherapy)	3 (50%)	1 (17%)	
Pembrolizumab (combined therapy)	0 (0%)	2 (33%)	
Duration of ICI administration to RT (months)	3 (2–17)	4 (2–11)	0.999
*Number of lesions*			0.455
1–5	2 (33%)	0 (0%)	
> 5	4 (66%)	6 (100%)	
*Purpose of RT*			0.999
Pain relief	3 (50%)	4 (66%)	
Reduction of lesions	2 (33%)	1 (17%)	
Improvement of stenosis	1 (17%)	1 (17%)	
*Irradiated tumour sites*			0.740
Bone	2 (33%)	4 (66%)	
Lymph node	3 (50%)	1 (17%)	
Others	1 (17%)	1 (17%)	
*Number of lesions for RT*			0.318
1	2 (33%)	5 (83%)	
2	2 (33%)	0 (0%)	
≥ 3	2 (33%)	1 (17%)	
PTV (cc)	135.6 (29.7–276.1)	99.3 (41.4–579.0)	0.699
*RT dose*			0.455
50 Gy/4 fx	1 (17%)	0 (0%)	
40–20 Gy/5 fx	5 (83%)	4 (66%)	
30 Gy/10 fx	0 (0%)	2 (33%)	
*RT method*			0.455
IMRT	6 (100%)	4 (66%)	
3DCRT	0 (0%)	2 (33%)	
*Median follow-up (years)*	1.8 (0.5–2.3)	0.9 (0.5–1.9)	0.240

Data are presented as median (range) or number (%)

*AE*  abscopal effect, *n*  total number of patients, *PD-L1*  programmed cell death ligand 1, *CD*   cluster of differentiation, *ICI*   immune checkpoint inhibitor, *RT*  radiotherapy, *PTV*  planning target volumes, *fx*   fractions, *IMRT*   intensity-modulated radiotherapy, *3DCRT*  three-dimensional conformal radiotherapy

**Table 2:** Details of case-specific PD-L1 expression and treatment

Cases	Primary sites	Pathology (carcinoma)	PD-L1 expression			Systemic therapy	RT dose	RT location	Number of lesions	AE
			Measurement lesion	Sampling method	Tumour proportion score					
1	Lung	Large cell	Primary (lung, NI)	Surgical specimens	≥ 50%	Pembrolizumab	30 Gy/5 fx	Lymph node	> 5	Yes
2	Lung	Adeno	Primary (lung, I)	Biopsy	≥ 50%	Pembrolizumab	35 Gy/5 fx	Lung	3	Yes
3	Lung	Adeno	Metastasis (lymph node, NI)	Surgical specimens	≥ 50%	Pembrolizumab	50 Gy/4 fx	Lymph node	3	Yes
4	HN	Squamous cell	Primary (nasal cavity, NI)	Biopsy	1%-49%	Nivolumab	25 Gy/5 fx	Lymph node	> 5	Yes
5	Stomach	Adeno	NA	NA	NA	Nivolumab	35 Gy/5 fx	Bone	> 5	Yes
6	Kidney	Clear cell	NA	NA	NA	Nivolumab + Ipilimumab	20 Gy/5 fx	Bone	> 5	Yes
7	HN	Squamous cell	Metastasis (liver, I)	Biopsy	1%-49%	Nivolumab	40 Gy/5 fx	Liver	> 5	No
8	Orbit	Melanoma	Metastasis (nasal cavity, NI)	Biopsy	< 1%	Nivolumab	30 Gy/5 fx	Bone	> 5	No
9	Kidney	Clear cell	Metastasis (lymph node, NI)	Surgical specimens	< 1%	Nivolumab	30 Gy/10 fx	Lymph node	> 5	No
10	Lung	Adeno	Primary (lung, NI)	Biopsy	< 1%	Pembrolizumab + Carboplatin + Pemetrexed	30 Gy/10 fx	Bone	> 5	No
11	Bladder	Transitional cell	NA	NA	NA	Pembrolizumab	30 Gy/5 fx	Bone	> 5	No
12	Kidney	Clear cell	NA	NA	NA	Pembrolizumab + Lenvatinib	35 Gy/5 fx	Bone	> 5	No

*HN*   Head and neck, *PD-L1*   programmed cell death ligand 1, *NI*  non-irradiated, *I*   irradiated, *NA*  not applicable, *RT*   radiotherapy, *AE*  abscopal effect

### Typical examples of the AE

#### Case 1

**Fig. 2:**
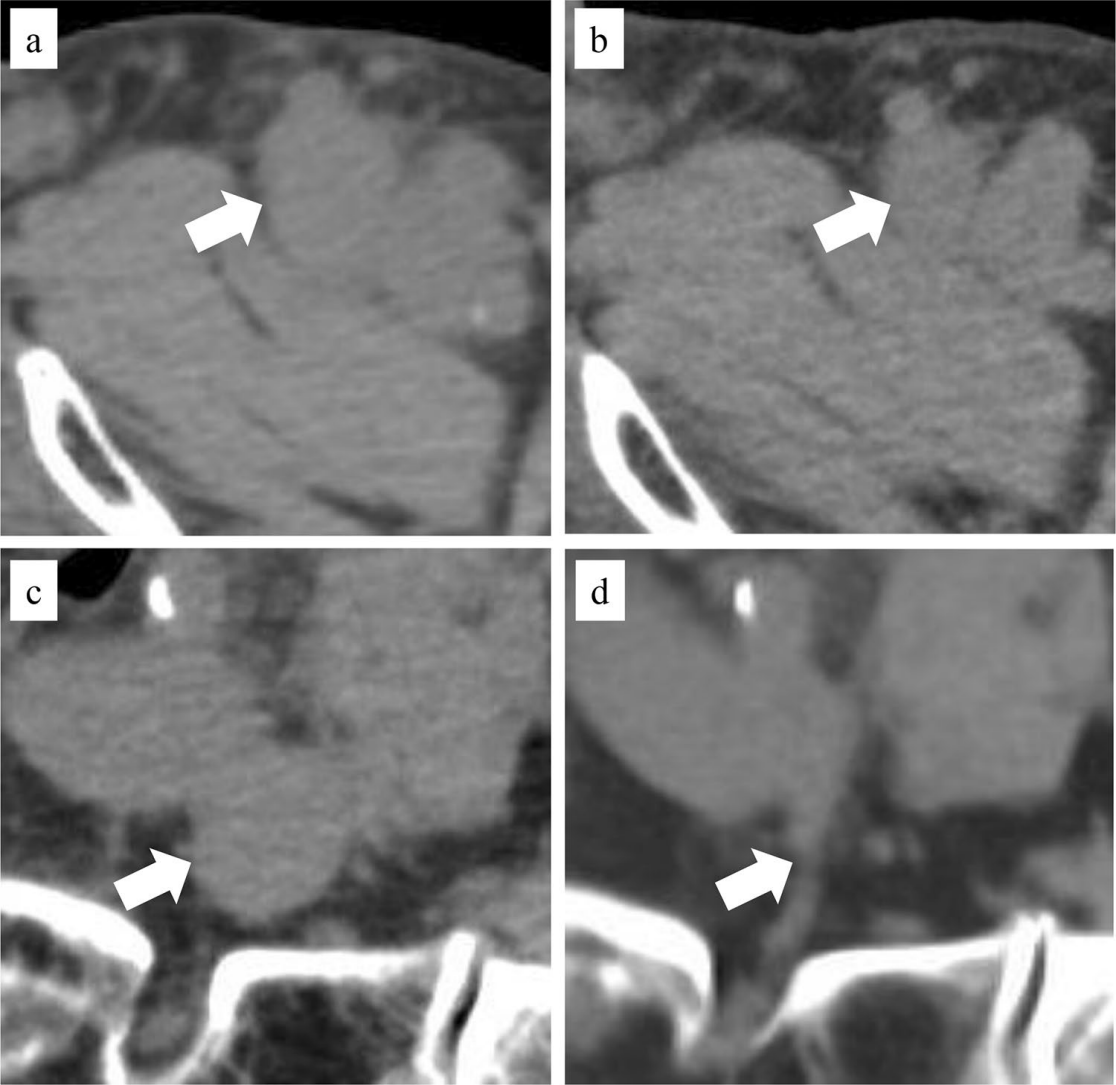
CT image of case 1. **a** Left inguinal lymph node before radiotherapy; **b** left inguinal lymph node 2 months after radiotherapy; **c** left iliac lymph node before radiotherapy (unirradiated assessment lesion); and **d** left iliac lymph node 4 months after radiotherapy (unirradiated assessment lesion). White arrow, tumour; *CT* computed tomography

An 83-year-old male patient was diagnosed with large-cell lung cancer. Owing to the presence of multiple distant metastases from the time of diagnosis, pembrolizumab (200 mg/body/3 weeks) was used as the first-line treatment. Four months after treatment initiation, the patient requested radiotherapy owing to increasing pain from the left inguinal lymph node metastasis despite a mild increase shown on imaging (Fig. 2a). Palliative radiotherapy was performed with 30 Gy/5 fractions. Two months later, the left inguinal lymph node metastasis decreased in size, and the pain disappeared (Fig. 2b). Pembrolizumab treatment continued, and another imaging scan 2 months later showed a marked reduction in unirradiated left iliac lymph nodes (Fig. 2c and d).

#### Case 2

**Fig. 3:**
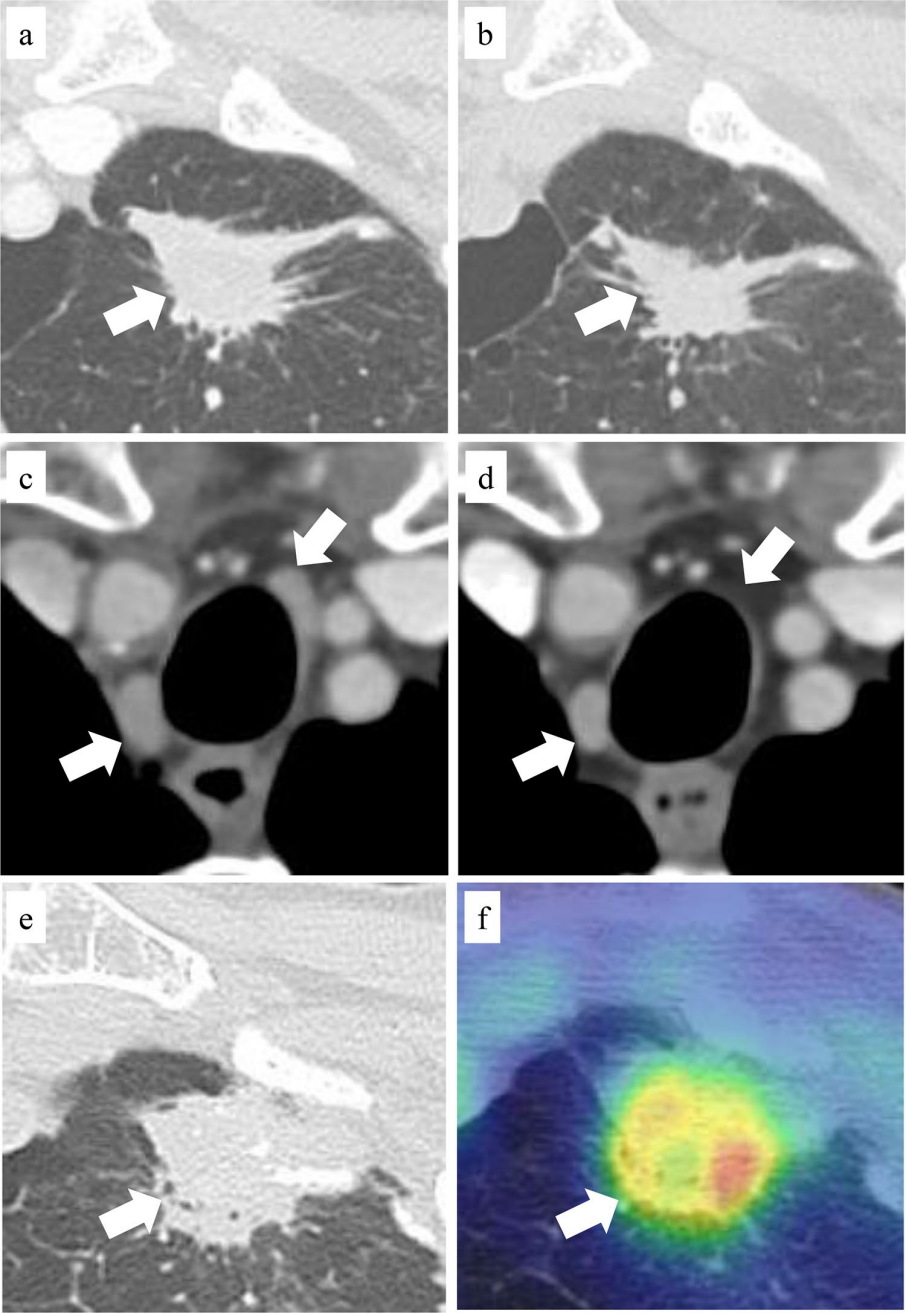
CT and PET/CT images of case 2. **a** Left primary lung lesion before radiotherapy; **b** left primary lung lesion 3 months after radiotherapy; **c** mediastinal lymph node before radiotherapy (unirradiated assessment lesion); **d** mediastinal lymph node 6 months after radiotherapy (unirradiated assessment lesion); **e** left primary lung lesion 1 year and 10 months after radiotherapy; and **f** left primary lung lesion 1 year and 10 months after radiotherapy (PET/CT). White arrow, tumour; *CT* computed tomography, *PET* positron emission tomography

An 82-year-old male patient was diagnosed with lung adenocarcinoma. He had brain metastases at diagnosis; therefore, pembrolizumab (200 mg/body/3 weeks) was used as a first-line treatment after stereotactic radiotherapy to the brain. Pembrolizumab was effective and continued long term; however, after 1 year, the tumours grew slowly, particularly in the primary left upper lobe lesion. One year and 5 months after the start of pembrolizumab treatment, radiotherapy was performed on the left primary lung lesion with 35 Gy/5 fractions to reduce the volume (Fig. 3a). Three months after irradiation, the primary lesion shrunk (Fig. 3b). Simultaneously, pembrolizumab treatment was discontinued because of the presence of grade 2 radiation pneumonitis, which resolved mildly after a short course of steroids. However, pembrolizumab treatment was not restarted and was terminated after a follow-up visit 3 months later because the non-irradiated mediastinal lymph node metastases and other lesions decreased or disappeared (Fig. 3c and d). One year and 10 months after irradiation, the primary lesion had enlarged again (Fig. 3e), and positron emission tomography revealed uptake (Fig. 3f). A thoracoscopic left upper quadrant resection was performed as no other noteworthy lesions were found, although recurrence was considered likely. However, the pathological results showed only necrosis and fibrosis with no malignancy, indicating that this was a pseudoprogression following radiotherapy. The patient was alive without treatment or progression on the last observation date (2 years and 3 months after irradiation).

### Secondary endpoint outcomes

**Fig. 4:**
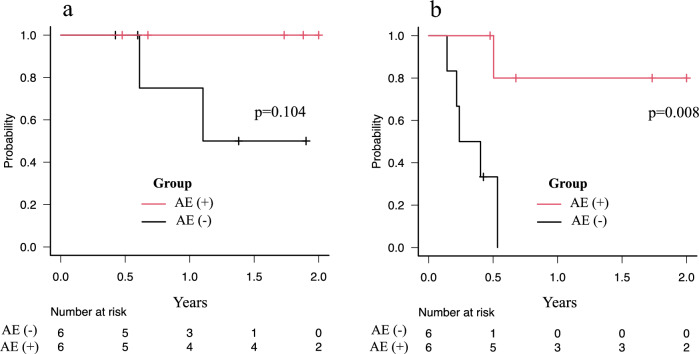
Kaplan–Meier curves for OS (**A**) and PFS (**B**) in the patient groups separated by AEs. *OS* overall survival, *PFS* progression-free survival, *AE* abscopal effect

All 12 (100%) patients completed radiotherapy. The number of responders at 3 months (median) post-irradiation was nine (75%; CR, one case; PR, eight cases). Palliative effects were achieved in all seven cases where radiotherapy was performed for pain relief. Survival curves are shown in Fig. 4. Patients in the AE group had a significantly better 1-year PFS than those in the non-AE group (80.0% vs 0.0%, *p* = 0.008). Although the difference was not significant, the 1-year OS was better in patients in the AE group (100.0% vs 75.0%, *p* = 0.104). Two patients in the non-AE group died during the last follow-up. Of the remaining 10 patients, two from the AE group were untreated and progression-free.

Grade 2 toxicity was observed in four (33%) patients: three in the AE group and one in the non-AE group. Two patients had radiotherapy-related pneumonia and oesophagitis, whereas the remaining two had ICI-related pneumonia and enteritis. No grade 3 or higher toxicities were observed in any category.

## Discussion

To the best of our knowledge, this is the first study to analyse the incidence of AEs as a primary endpoint when irradiating Japanese patients with oligoprogressive tumours during ICI therapy. In six of the 12 (50%) patients, AEs were identified, implying that lesions outside the radiation field showed volume reduction. Patients in the AE group had a significantly better 1-year PFS than those in the non-AE group (*p* = 0.008), and two patients were untreated, with PFS, at the end of follow-up. No grade 3 or higher toxicities were found during the observation period.

Although the role of radiotherapy in advanced cancer has been limited to palliation, its indications have expanded recently. Oligometastatic disease, which generally presents with fewer than five metastases, is a prime candidate [17, 18]. Radiotherapy for oligometastatic disease is largely beneficial for improving prognosis [19–21]. Recently, oligoprogressive disease has gained attention as the next target for radiotherapy. Kim et al. conducted a systematic review of eight prospective trials on the local treatment of oligoprogressive disease [14]. An analysis of 290 patients showed that there may be a benefit of adding local treatment to systemic treatment for lung, prostate and kidney cancers. Particularly, a study on non-small cell lung cancer demonstrated that stereotactic radiotherapy improved PFS and OS compared to systemic therapy alone. Another study focused on the combination of ICI and radiotherapy in patients with oligoprogressive disease. Chicas-Sett et al. conducted an observational study of stereotactic radiotherapy in patients with metastatic non-small cell lung cancer or melanoma that progressed despite anti-PD-1 antibodies [22]. The primary endpoint in 50 patients was an objective response rate of 42%, with a good median PFS (14.2 months) and median OS after stereotactic body radiation therapy (37.4 months). Our results are similar to those of their report in that the AEs of radiotherapy improved PFS and played a role in delaying the new systemic treatment line. We believe that our results will help formulate hypotheses for future randomised controlled trials of oligoprogressive disease.

Although this study showed an AE rate of 50%, it was difficult to determine whether this number was high, as the definition of the AE varies slightly from study to study and is not well established. Previous studies reported considerable heterogeneity regarding design, inclusion criteria and treatment variables [23–25]. This point is reported in detail with historical background as drawbacks in defining AEs by Demaria et al. [26]. We should be particularly careful when interpreting trials where ICI and radiotherapy are concurrently initiated [25, 27]. In this case, lesion reduction outside the irradiated field may reflect the effect of ICI therapy and thus overestimate the AEs. Demaria et al. advocate for a combination with radiotherapy in patients who do not respond to immunotherapy alone as one approach to correctly target AEs [26]. In this study, the rate of AE was assessed accordingly. Taking this important issue into account, our survey of previous reports of combined ICI and radiotherapy showed a median AE rate of approximately 25% (range 0–65%) [9–11, 22, 28–30]. Therefore, we believe that the oligoprogressive diseases on which we focused are relatively promising targets for the AE.

Combining another predictor with oligoprogressive disease status may further increase the expected AE value. Numerous potential predictors have been addressed in previous reports [31]. However, predictors of the AE have not been established, and no statistically significant factors were found in this study. Although a retrospective study reported that lymphocyte count is a predictor of AEs, this study did not show the same result [32]. We interpret this as lymphocyte counts being susceptible to other factors, such as infection. A few previous studies have focused on PD-L1 expression and AEs. For example, Yaguchi et al. reported that a patient with lung cancer who was strongly positive for PD-L1 had almost complete resolution of lesions, including non-irradiated areas, after radiotherapy and anti-PD-1 antibodies [33]. Notably, in our study, all three strongly positive patients also experienced AEs, and all three negative patients experienced no AEs, although this indicated no significant difference. Some scholars have argued that PD-L1 expression alone has limitations in successfully predicting, as it may also indicate T-cell exhaustion and thus reduced systemic efficacy [34]. Therefore, further research is required to establish predictive factors.

Our study was limited by its non-interventional nature, lack of a control group and small sample size. Notably, this study’s definition of oligoprogressive disease did not always correspond to that used in previous studies. Recently, the American Radium Society suggested a definition of oligoprogression as ≤ 3 discrete areas of progression in non-small cell lung cancer in prior or ongoing systemic therapy [13]. Although it may require considerable time to reach a consensus on definitions for all cancer types and to coordinate opinions with other associations, future clinical trials should consider these recommendations. Another limitation is the possibility of including pseudoprogression after ICI treatment in eligible patients. Pseudoprogression after ICI administration occurs in a small percentage of cases, sometimes with a delayed onset of shrinkage after several months [35]. Although this study enrolled patients > 2 months after the initiation of ICI therapy for compatibility with iRECIST [36], we recognised that pseudoprogression is a limitation that cannot be excluded in AE studies.

In conclusion, irradiation of solid tumours showing oligoprogression during ongoing ICI therapy appears promising, as AEs were expected in 50% of the patients. Patients with AEs show improved PFS and sometimes achieve long-term PFS after the completion of ICI therapy. We believe that our results advance the study of AEs and provide a cornerstone for improving the prognosis of patients with advanced cancers.
